# Reproducibility of a quantitative system for assessing the quality of diagnostic ultrasound

**DOI:** 10.1590/0100-3984.2017.0021

**Published:** 2018

**Authors:** Wagner Iared, Andrea Puchnick, Eduardo Bancovsky, Paulo Roberto Bettini, Leonardo Modesti Vedolin, Maria Cristina Chammas

**Affiliations:** 1 MD, PhD, Radiologist, Advisor for the Graduate Program in Evidence-Based Medicine of the Escola Paulista de Medicina da Universidade Federal de São Paulo (EPM-Unifesp), São Paulo, SP, Brazil.; 2 Professor, Coordinator of Instruction and Research in the Department of Diagnostic Imaging of the Escola Paulista de Medicina da Universidade Federal de São Paulo (EPM-Unifesp), São Paulo, SP, Brazil.; 3 MD, Radiologist, Coordinator of the Ultrasound Division of Diagnósticos da América S/A, Barueri, SP, Brazil.; 4 MD, PhD, Neuroradiologist, Director of Radiology and Diagnostic Imaging at Diagnósticos da América S/A, Barueri, SP, Brazil.; 5 MD, PhD, Radiologist, Director of the Ultrasound Department of the Instituto de Radiologia do Hospital das Clínicas da Faculdade de Medicina da Universidade de São Paulo (InRad/HC-FMUSP), São Paulo, SP, Brazil.

**Keywords:** Ultrasonography, Quality control, Quality improvement, Certification, Accreditation

## Abstract

**Objective:**

To present a quantitative system for assessing the quality of ultrasound examinations-SQUALUS-and to determine its reproducibility, taking into consideration the images on file, as well as the consistency between the images obtained and the final report.

**Materials and Methods:**

The system includes questions related to the number of images; the appropriateness of images in relation to the protocol established; focus adjustment; depth; gain; and appropriateness of the measurements for B-mode examinations. For Doppler examinations, the system includes questions related to the appropriateness of color images, the spectral analysis, and correction of the insonation angle. To assess the quality of the report, the system includes questions related to the consistency between the images obtained and the contents of the report. An overall numerical score was assigned by averaging the scores for image quality and for the contents of the report. Two independent examiners, each blinded to the evaluation of the other, assessed 30 different types of ultrasound examinations.

**Results:**

There was statistically significant agreement between the two examiners for 8 of the 10 questions related to image quality. For the questions related to the quality of the reports, the interexaminer agreement was almost perfect.

**Conclusion:**

The proposed quantitative system for assessing the quality of ultrasound examinations is a reproducible tool that can be used in audits and accreditation programs.

## INTRODUCTION

There is real concern about the quality of medical services in Brazil. The National Health Insurance Agency of the Brazilian National Ministry of Health has developed norms and standards, such as the Program for the Dissemination of Health Insurance Provider Qualifications, established by Normative Resolution no. 267 of August 24, 2011, and the Program for Monitoring the Quality of Health Insurance Providers, established by Normative Resolution no. 275 of November 1, 2011^(^^1^^)^. Those programs promote the quality of providers focused on beneficiaries. The programs are also in line with the efforts of the Colégio Brasileiro de Radiologia e Diagnóstico por Imagem (CBR, Brazilian College of Radiology and Diagnostic Imaging) in its Programa de Acreditação em Diagnósticos por Imagem (PADI, Program for the Accreditation of Diagnostic Imaging Clinics), as well as in its quality certification programs^(^^2^^,^^3^^)^.

Quality assessments are performed differently for ultrasound examinations than for other diagnostic imaging examinations. In modalities such as mammography, radiography, computed tomography, and magnetic resonance imaging, non-medical professionals (technicians, technologists, and biomedical professionals) acquire and register images according to certain protocols, which can be altered on a case-by-case basis by radiologists. In ultrasound, however, it is the primary care physician who acquires the images and prepares the final report. Although the CBR, through the Comissão Nacional de Ultrassonografia (CNUS, National Ultrasound Commission) and the PADI^(^^2^^,^^3^^)^, together with other institutions, has standardized protocols for minimum documentation of the images obtained in each type of ultrasound examination, the results vary markedly among different physicians and facilities.

With respect to the ultrasound report, unlike other imaging modalities, in which the same images can be interpreted by more than one radiologist, ultrasound is a dynamic method in which the scanning of the various organs and structures must be performed in several planes, although only a limited number of images are recorded in the documentation. It is presumed that the images recorded are the most representative of the normal and pathological findings, according to what is specified in the minimum documentation protocols. This makes it difficult to evaluate the technical quality of ultrasound examinations.

To date, there have been no studies suggesting any method of evaluating the quality of ultrasound examinations similar to the one we are proposing. Although there have been studies focusing on the technical aspects of the equipment^(^^4^^,^^5^^)^, the technical parameters related to training methods^(^^6^^)^, and the influence of image quality on specific diagnoses^(^^7^^-^^9^^)^, there have been none focusing on the quality of the examination performed and reported by medical professionals.

The objective of this work is to present and determine the reproducibility of a quantitative system of quality evaluation of ultrasound examinations-the Sistema Quantitativo de Avaliação da Qualidade de Exames de Ultrassonografia (SQUALUS, Quantitative System for Assessing the Quality of Ultrasound Examinations)-taking into account the images on file and the concordance between the images obtained and the final report.

## MATERIALS AND METHODS

### Development of criteria for evaluation of image quality

To evaluate the examinations, we used a checklist based on the PADI and CNUS/CBR regulations^(^^2^^,^^3^^)^, taking into consideration the technical parameters proposed by the American College of Radiology and the American Institute of Ultrasound in Medicine^(^^10^^)^.

For routine examinations, the following parameters were used:

- Minimum recommended number of images.
- Structures documented in accordance with the recommendations.
- Appropriate depth.
- Focus adjustment.
- Adjustment of transducer gain/frequency/gray-scale map
- Check of the appropriateness of the pertinent measures.

For Doppler examinations, the following were also evaluated:

- Appropriateness of color Doppler images.
- Appropriate spectral analysis.
- Angle correction for the analysis of flow velocities.

For each item, the evaluator should check and answer yes, no, or not applicable. For different types of examination, different weights were checked for each item. Table 1 shows the checklist used for all types of examinations, and Table 2 shows the weights of each question for the different types of examination. If the answer was yes for all questions, the total score would be 10. If an examination was totally non-standard-that is, the answer was no for all questions-the total score would be 0.

**Table 1:** Checklist for examination quality evaluation.

	Checklist
1	Is the number of images on file equal to or greater than the recom-mended minimum for the type of examination?
2	Have the structures relevant to the examination been documented in the recommended incidences?
3	Does the depth used allow the best visualization of the structures of interest?
4	Was care taken to focus on the center of the structures of interest?
5	Was the gain adjusted so as to obtain the best contrast between the anatomical structures in the images obtained?
6	Have the recommended measurements for the type of examination been appropriately carried out?
7	Have all structures or changes of interest been documented with color Doppler mapping?
8	Was spectral analysis of the relevant vessels performed appropriately?
9	Were flow velocity measurements performed with appropriate angle correction?

**Table 2:** Weights for the questions according to the type of examination evaluated.

Type of examination	Adequate number of photos?	Appropriate structures documented?	Adequate depth?	Focus on the center of the structure?	Adequate gain?	Appropriate measurements?	Appropriate color documentation?	Appropriate spectral analysis?	Appropriate Doppler angle correction?
US examination without Doppler	2	3	1	1	1	2	N/A	N/A	N/A
Transvaginal pelvic Doppler US	1	2	1	1	1	2	1	1	N/A
Doppler US of the scrotum	1	2	1	1	1	2	1	1	N/A
Doppler US of the portal system	1	1	1	1	1	1	1	1	2
Renal Doppler US	1	1	1	1	1	1	1	1	2
Doppler US of thyroid nodule(s)	1	1	1	1	1	2	2	1	N/A
Doppler US of diffuse thyroid disease	1	1	1	1	1	1	1	1	2
Obstetric Doppler US	1	1	1	1	1	1	1	1	2
Doppler US of the peripheral arteries	1	1	1	1	1	1	1	1	2
Doppler US of the carotid artery	1	1	1	1	1	1	1	1	2
Venous Doppler US	1	1	1	1	1	2	1	2	N/A

N/A, not applicable.

### Development of criteria for evaluating the appropriateness of the report and its concordance with the images

Because imaging is operator-dependent, the final report of the ultrasound examination was evaluated according to its concordance with the images obtained. The degree of agreement between the report and the images was classified into four different categories:

- The report is appropriate and consistent with the images.
- There is disagreement, but the disagreement is of little relevance.
- There is disagreement of moderate relevance.
- There is highly relevant disagreement.

Minor typographical errors and spelling mistakes that did not compromise the understanding of the report and the final diagnosis were classified as inconsistencies of little relevance. Gross errors in the text, inappropriate descriptions, and dubious diagnostic impressions were classified as moderately relevant disagreements. Descriptions and diagnostic impressions inconsistent with the images on file were classified as highly relevant disagreements. Appropriate, consistent reports were given a score of 10; reports containing inconsistencies of little relevance were given a score of 7; reports with moderately relevant disagreements were given a score of 3; and reports with highly relevant disagreements were given a score of 0.

The report and the images are inseparable, so the arithmetic mean between the quality score assigned to the images and the score for the appropriateness/consistency of the report was used as the final parameter to judge the quality of each examination evaluated. If an examination presented excellent image quality and the report had highly relevant disagreements, the final combined score was 5. If the images were excellent and the report had moderately relevant disagreements, the final combined score was 6.5. If the report was appropriate and consistent but the images did not meet any of the recommended criteria, the final combined score was 5.

### Concordance test

Prior to performing the examinations, the medical team was informed of the documentation protocol to be followed and was made aware of the fact that the examinations would be randomly audited. The identity of the physicians who performed the examinations involved in this study, as well as the identity of the patients, was kept confidential.

Two independent evaluators, both CBR-certified radiologists, with 23 and 16 years of experience in ultrasound, respectively, evaluated the same 5 examinations of each type, for a total of 30 evaluations per evaluator. The examinations evaluated were randomly extracted from those performed in the laboratories of a private company in São Paulo, between August and September of 2016. Each evaluator applied the checklist and was blinded to the evaluation of the other. The types of examinations evaluated were as follows: total abdominal ultrasound; transvaginal pelvic ultrasound; ultrasound of the breasts; Doppler ultrasound of thyroid nodules; Doppler ultrasound of the thyroid for evaluation of diffuse disease; Doppler ultrasound of the carotid artery; and Doppler ultrasound of the veins of the lower limbs.

### Statistical analysis

The data collected in this study were initially submitted to descriptive statistical analysis. For the quantitative (continuous) variables "image score", "report score", and "mean final examination score", we calculated summary measures, such as mean and standard deviation. Qualitative (categorical) variables were analyzed by calculating absolute and relative frequencies.

For all of the parameters evaluated, the agreement between the two evaluators was quantified by calculating the kappa statistic (k). To quantify the agreement between the scores and means obtained by the two evaluators, a Bland-Altman plot was constructed, after which the intraclass correlation coefficient (k_icc_) was estimated. For all measurements, 95% confidence intervals were calculated. The strength of the agreement, based on the kappa coefficients (k;k_icc_), was interpreted as shown in Table 3.

**Table 3:** Categorization of the strength of the agreement, based on the kappa coefficients (κ; κ_icc_).

κ; κ_icc_	Strength of the agreement
< 0.00	Less than chance
0.00-0.19	Poor
0.20-0.39	Weak
0.40-0.59	Moderate
0.60-0.79	Strong
0.80-0.99	Near-perfect
1.00	Perfect

Statistical evaluation was performed with the Statistical Package for the Social Sciences, version 16.0 for Windows (SPSS Inc., Chicago, IL, USA). For all conclusions reached by inferential analysis, the level of significance was set at 5%.

## RESULTS

The evaluators presented statistically significant agreement for 8 of the 10 questions (Table 4). The strength of agreement ranged from perfect to poor.

**Table 4:** Agreement between evaluators 1 and 2 for the quality criteria.

	Evaluator 1			Evaluator 2				
Quality criteria	N	(%)		N	(%)	κ [95% CI]	Force of agreement	*P*
Adequate number of photos						0.783 [0.574-0.992]	Important	< 0.0001
Yes	27	(90.0)		28	(93.3)			
No	3	(10.0)		2	(6.7)			
Appropriate structures documented						0.760 [0.598-0.922]	Important	< 0.0001
Yes	25	(83.3)		25	(83.3)			
No	5	(16.7)		5	(16.7)			
Adequate depth						0.000 [-0.181-0.181]	Bad	1.000
Yes	24	(80.0)		25	(83.3)			
No	6	(20.0)		5	(16.7)			
Focus on the center of the structure						0.524 [0.319-0.729]	Moderate	0.003
Yes	26	(86.7)		24	(80.0)			
No	4	(13.3)		6	(20.0)			
Adequate gain						0.047 [0.014-0.080]	Bad	0.786
Yes	29	(96.7)		28	(93.3)			
No	1	(33.3)		2	(6.7)			
Appropriate measurements						0.760 [0.598-0.922]	Important	< 0.0001
Yes	26	(86.7)		25	(83.3)			
No	4	(13.3)		5	(16.7)			
Appropriate color photos						1.000 [-]	Perfect	< 0.0001
Yes	15	(50.0		15	(50.0)			
Not applicable	15	(50.0)		15	(50.0)			
Appropriate spectral analysis						1.000 [-]	Perfect	< 0.0001
Yes	10	(33.3)		10	(33.3)			
No	5	(16.7)		5	(16.7)			
Not applicable	15	(50.0)		15	(50.0)			
Appropriate Doppler angle correction						1.000 [-]	Perfect	< 0.0001
Yes	5	(16.7)		5	(16.7)			
No	2	(6.7)		2	(6.7)			
Not applicable	23	(76.7)		23	(76.7)			
Concordance with the report								
Good	22	(73.3)		22	(73.3)	0.769 [0.665-0.873]	Important	< 0.0001
Mild disagreement	5	(16.7)		4	(13.3)			
Moderate disagreement	2	(6.7)		3	(10.0)			
Significant disagreement	1	(33.3)		1	(33.3)			

95% CI, 95% confidence interval.

**Figure 1:**
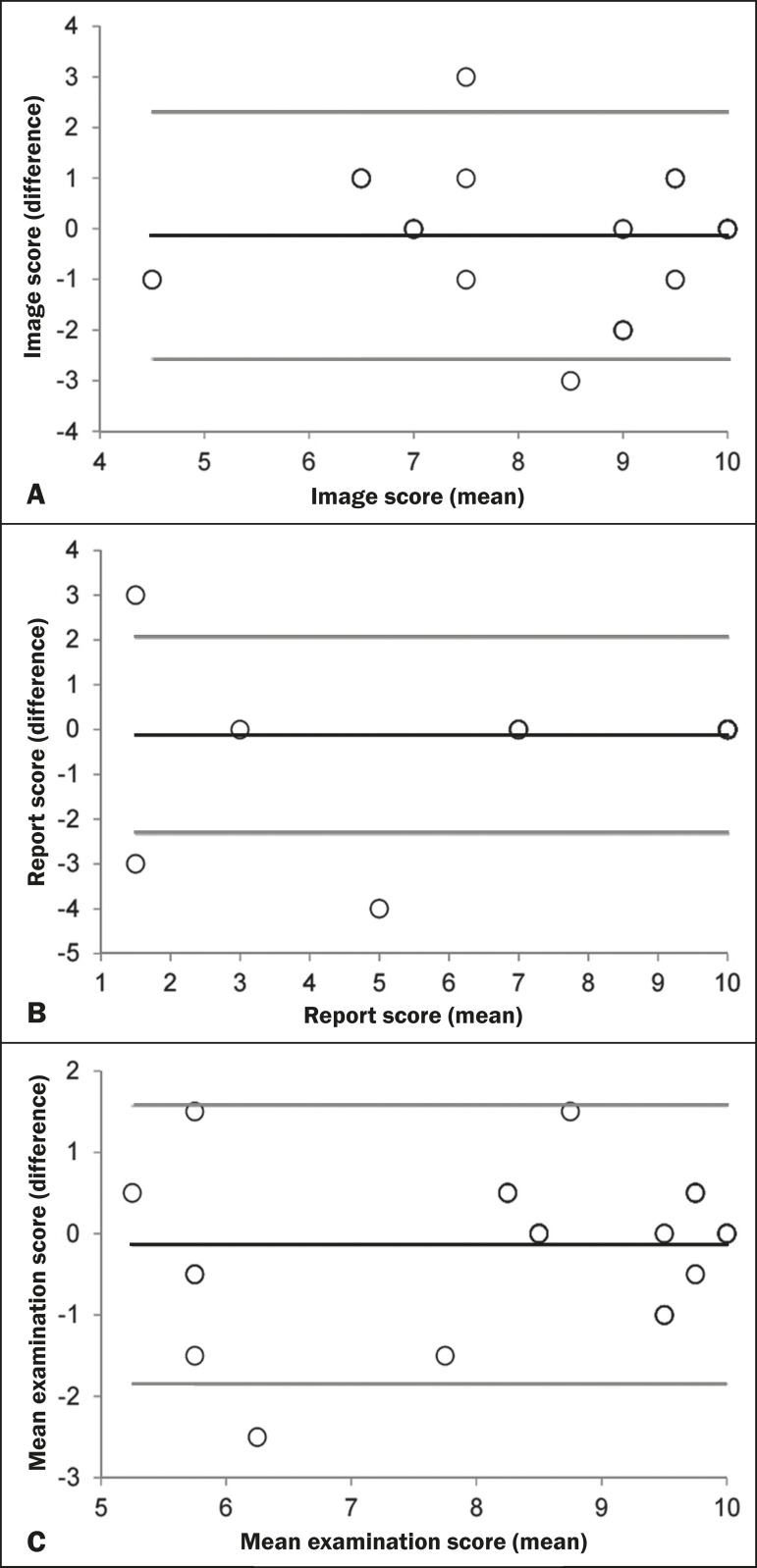
Distribution of the scores for the images and the reports, together with the final mean scores, given for the examinations by evaluators 1 and 2. The mean of the difference between the two evaluators was -0.1 ± 1.2 in relation to the image scores (**A**), -0.1 ± 1.1 in relation to the report scores (**B**) and -0.1 ± 0.9 compared with the final mean examination score (**C**). Black line: mean of the differences. Gray lines: 95% confidence intervals.

In relation to the reproducibility of the image scores, report scores, and final means, the differences between the analyses of evaluator 1 and those of evaluator 2 are shown in the Bland-Altman plot (Figure 1). Overall, in the three parameters analyzed, the evaluators presented very similar values (mean differences varying between −0.1 ± 0.9 and −0.1 ± 1.2), indicating almost perfect agreement (Table 5).

**Table 5:** Reproducibility scores for images and reports, together with the final mean scores, given by evaluators 1 and 2.

	Evaluator 1		Evaluator 2			
Parameter evaluated	Mean ± SD		Mean ± SD	κ_icc_ [95% CI]	Force of agreement	*P*
Image score	8.5 ± 1.8		8.4 ± 1.7	0.861 [0.707-0.934]	Almost perfect	< 0.0001
Report score	8.7 ± 2.6		8.6 ± 2.8	0.958 [0.911-0.980]	Almost perfect	< 0.0001
Final mean score	8.6 ± 1.5		8.5 ± 1.7	0.926 [0.844-0.965]	Almost perfect	< 0.0001

SD, standard deviation; 95% CI, 95% confidence interval.

## DISCUSSION

The almost perfect agreement between the evaluators for the mean image quality scores, the report scores, and the final mean scores of the examinations demonstrates that the method is reproducible. For the checklist criteria with the greatest relevance for the final mean score, the agreement was classified as significant and perfect. Although depth, focus on the center of the structure, and gain were less concordant, these data did not significantly affect the final mean score, because each of those criteria had a weight of only one point in the mean for all types of examinations.

The ideal form of evaluation of the quality of ultrasound examinations would be through a retrospective analysis, correlating their results with the clinical follow-up and eventual findings on other imaging tests, as well as with surgical and pathological findings. Such an evaluation method may be applicable in the hospital environment, where integration between radiologists and teams of other specialties facilitates the monitoring and favorable evolution of cases. The examinations performed on an outpatient basis do not have the benefit of that type of confirmation, being limited to the evaluation of the person who performs them.

The quality of the photographic documentation of ultrasound examinations, following established documentation protocols, should not be considered mere whim, knowing that in some cases excellent physicians perform accurate diagnoses with the method, although without recording the established patterns. The observation of such protocols, in addition to serving as an eventual legal support, proves that the examination was carried out, exhausting, from a technical point of view, the method in question. Examinations with poorly documented images are classified as poor quality examinations.

Adequate photographic documentation demonstrates that a refined technique was used, which confers significantly greater sensitivity on the method, proving that scans were performed in different planes of the studied organs. The adjustment of depth and focus optimizes the detection of lesions and the detailing of their characteristics.

The evaluated parameters cover the number of images, the appropriateness of the images to the established protocol, the care in the adjustment of the focus, depth and gain, and adequacy of the measurements, for the examinations in mode B. For Doppler examinations, we evaluated the appropriateness of the color photos, spectral analysis, and angle correction. In the SQUALUS method, the first two parameters evaluated are precisely to determine whether the examination followed the minimum documentation guidelines. The weight for each criterion was assigned in a consensus meeting of the authors, aiming at valuing the essential aspects of each type of examination. That was the most time-consuming phase of the study.

The final quantitative result allows the accrediting entity to define a cut-off point from which the examination is considered acceptable. Our suggestion is that examinations with a final mean score of 7 or higher be considered qualified, because the reports are consistent or contain only inconsistencies of little relevance, which do not imply disagreement with the final diagnosis. With scores 0 and 3 for disagreements of high or moderate relevance, respectively, these cases would never reach a mean score of 7, even if the image quality score was 10. Likewise, appropriate reports with an image quality score lower than 4 would not reach the mean score of 7. In our experience, in poorly documented examinations, the evaluators rarely find good agreement between the images and the report. However, we must emphasize, that this study is limited by the small number of examinations of each type, which made it impossible to assess agreement for each of them.

For examinations performed in urgent and emergency situations, limited documentation or even a report without documented images is acceptable. However, without proper recording of images, it is impossible for the quality of the final result to be validated through audits conducted by the facility itself or by accreditation programs. Therefore, when submitting examinations to accreditation programs, care should be taken to follow the minimum protocols of photographic documentation required.

## CONCLUSION

The SQUALUS is a reproducible tool that can be used in audits and accreditation programs.
